# STING contributes to anti-glioma immunity via triggering type-I IFN signals in the tumor microenvironment

**DOI:** 10.1186/2051-1426-2-S3-P228

**Published:** 2014-11-06

**Authors:** Takayuki Ohkuri, Arundhati Ghosh, Akemi Kosaka, Jianzhong Zhu, Maki Ikeura, Michael David, Simon C Watkins, Saumendra N Sarkar, Hideho Okada

**Affiliations:** 1 Department of Neurological Surgery, University of Pittsburgh School of Medicine, Pittsburgh, PA; 2 Department of Surgery, University of Pittsburgh School of Medicine, Pittsburgh, PA; 3 Department of Immunology, University of Pittsburgh School of Medicine, Pittsburgh, PA; 4 Department of Microbiology and Molecular Genetics, University of Pittsburgh School of Medicine, Pittsburgh, PA; 5 Department of Cell Biology and Physiology, University of Pittsburgh School of Medicine, Pittsburgh, PA; 6 Department of Brain Tumor, University of Pittsburgh School of Medicine, Pittsburgh, PA; 7 Cancer Immunology, University of Pittsburgh Cancer Institute, Pittsburgh, PA; 8 Cancer Virology Programs, University of Pittsburgh Cancer Institute, Pittsburgh, PA; 9 Division of Biology, UCSD, La Jolla, CA

## 

**Figure 1:**
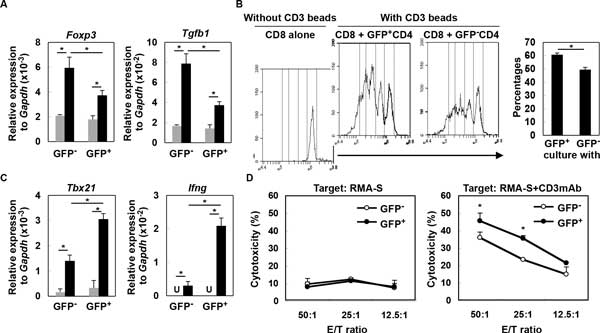
**Type-I IFNs directly impact on T-cell functions in glioma-developing mice**. (A) CD4^+ ^cells from draining LN derived from glioma-developing tdTomato mice were sorted into GFP^- ^or GFP^+ ^cells and incubated with (black bars) or without (grey bars) anti-CD3mAb. After 4 h, total RNA was extracted for evaluation of *Foxp3 *and *Tgfb1 *mRNA levels by qRT-PCR. (B) CFSE-labeled WT CD8^+ ^T-cells were co-cultured with GFP^- ^or GFP^+ ^CD4^+ ^T-cells in the presence of CD3 beads. After 60 h, division of CFSE-labeled CD8^+ ^T-cells gated by reactivity to PE-Cy7-condjugated anti-CD8mAb was evaluated by CFSE intensity. As a negative control, CFSE-labeled WT CD8^+ ^T-cells were cultured without any stimulation (left panel). Histograms are representative of two independent experiments. The bar graph shows the percentage of CD8^+ ^cells that have divided at least twice in each of two stimulation conditions (N = 4/group; *p < 0.05). (C) GFP^- ^or GFP^+ ^CD8^+ ^T-cells were incubated with (black bar) or without (grey bar) anti-CD3mAb. After 4 h, total RNA was extracted for evaluation of *Tbx21 *and *Ifng *mRNA expression levels by qRT-PCR (U: undetected). (D) Cytotoxic activity of GFP^- ^and GFP^+ ^CD8^+ ^T-cells was evaluated by ^51^Cr-release assay. RMA-S cells untreated (left panel) or pretreated (right panel) with anti-CD3mAb (10 g/mL) were used as target cells. *p < 0.05 compared at the same E/T ratio.

While type-I interferons (IFNs) play critical roles in antiviral and antitumor activity, it remains to be elucidated how type-I IFNs are produced in sterile conditions of the tumor microenvironment and directly impacts tumor-infiltrating immune cells. We report that both human and *de novo *mouse gliomas show increased expression of type-I IFN messages, and in mice, CD11b^+ ^brain-infiltrating leukocytes (BILs) are the main source of type-I IFNs that is induced partially in a STING (stimulator of IFN genes)-dependent manner. Consequently, glioma-bearing *Sting^Gt/Gt ^*mice showed shorter survival, and lower expression levels of *Ifns *compared with wild-type mice. Furthermore, BILs of *Sting^Gt/Gt ^*mice show increased CD11b^+ ^Gr-1^+ ^immature myeloid suppressor and CD25^+ ^Foxp3^+ ^regulatory T (Treg) cells, while decreased IFN-γ-producing CD8^+ ^T cells. To determine the effects of type-I IFN expression in the glioma microenvironment, we utilized a novel reporter mouse model, in which the type-I IFN signaling induces the *Mx1 *(IFN-induced GTP-binding protein) promoter-driven Cre recombinase, which turns the expression of *loxp*-flanked *tdTomato *off, and turns green fluorescence protein (GFP) expression on, thereby enabling us to monitor the induction and effects of IFN signaling in the glioma microenvironment. CD4^+ ^T cells that received direct type-I IFN signals (i.e., GFP^+ ^cells) demonstrate lesser degrees of regulatory activity based on lower *Foxp3 *and *Tgfb1 *expression levels (Figure 1) as well as lesser suppression of CD8^+ ^T cell proliferation (Figure B). IFN-sensed CD8^+ ^T cells exhibit enhanced levels of Th1 markers, *Tbx21 *and *Igfng *(Figure C), as well as cytotoxic T-cell activity based on reverse antibody-dependent T-cell-mediated cytotoxicity assay (Figure D). Finally, intratumoral administration of a STING agonist (cyclic diguanylate monophosphate; c-di-GMP) improves the survival of glioma-bearing mice associated with enhanced type-I IFN signaling, *Cxcl10 *and *Ccl5 *and T cell migration into the brain. In a combination with subcutaneous OVA peptide-vaccination, c-di-GMP increased OVA-specific cytotoxicity of BILs and prolonged the survival. These data demonstrate significant contributions of STING to antitumor immunity via enhancement of the type-I IFN signaling in the tumor microenvironment, and imply a potential use of STING agonists for development of effective immunotherapy, such as the combination with antigen-specific vaccinations.

